# Home-Applied Dual-Light Photodynamic Therapy in the Treatment of Stable Chronic Periodontitis (HOPE-CP)—Three-Month Interim Results

**DOI:** 10.3390/dj10110206

**Published:** 2022-11-02

**Authors:** Saila Pakarinen, Riitta K. T. Saarela, Hannamari Välimaa, Anna Maria Heikkinen, Esko Kankuri, Marja Noponen, Heikki Alapulli, Taina Tervahartiala, Ismo T. Räisänen, Timo Sorsa, Tommi Pätilä

**Affiliations:** 1 Degree Program of Oral Hygiene, Metropolia University of Applied Sciences, 00920 Helsinki, Finland; 2 Department of Oral Health, Health and Social Services, 00530 Helsinki, Finland; 3 Department of Oral and Maxillofacial Diseases, Faculty of Medicine, University of Helsinki, Helsinki University Hospital, 00290 Helsinki, Finland; 4 Faculty of Medicine and Health Technology, University of Tampere, 33520 Tampere, Finland; 5 Department of Pharmacology, Helsinki University, 00100 Helsinki, Finland; 6 Department of Pediatric Dentistry, New Children’s Hospital, University of Helsinki, 00290 Helsinki, Finland; 7 Division of Oral Diseases, Department of Dental Medicine, Karolinska Institutet, 14152 Huddinge, Sweden; 8 Department of Congenital Heart Surgery and Organ Transplantation, New Children’s Hospital, University of Helsinki, 00290 Helsinki, Finland

**Keywords:** antibacterial photodynamic therapy, oral hygiene, periodontitis

## Abstract

A single-site, randomized clinical trial was designed to determine the efficacy of regular home use of Lumoral^®^ dual-light antibacterial aPDT in periodontitis patients. For the study, 200 patients were randomized to receive non-surgical periodontal treatment (NSPT), including standardized hygiene instructions and electric toothbrush, scaling and root planing, or NSPT with adjunctive Lumoral^®^ treatment. A complete clinical intraoral examination was conducted in the beginning, at three months, and at six months. This report presents the three-month results of the first 59 consecutive randomized subjects. At three months, bleeding on probing (BOP) was lower in the NSPT + Lumoral^®^-group than in the NSPT group (*p* = 0.045), and more patients in the NSPT + Lumoral^®^-group had their BOP below 10% (54% vs. 22%, respectively, *p* = 0.008). In addition, patients in the NSPT + Lumoral^®^-group improved their oral hygiene by visible-plaque-index (*p* = 0.0003), while the NSPT group showed no statistical improvement compared to the baseline. Both groups significantly reduced the number of deep periodontal pockets, but more patients with a reduction in their deep pocket number were found in the NSPT + Lumoral^®^ group (92% vs. 63%, *p* = 0.02). Patients whose number of deep pockets was reduced by 50% or more were also more frequent in the NSPT + Lumoral^®^-group (71% vs. 33%, *p* = 0.01). Patients with initially less than ten deep pockets had fewer deep pockets at the three-month follow-up in the Lumoral^®^ group (*p* = 0.01). In conclusion, adjunctive use of Lumoral^®^ in NSPT results in improved treatment outcomes at three months post-therapy.

## 1. Introduction

Periodontitis is a chronic inflammatory condition of the attachment and supporting tissues of the teeth. The subgingival microbiota and the host’s immune system are the most critical factors in its generation mechanism, leading to the irreversible destruction of soft and hard tissue in the periodontium [1].

Antibacterial photodynamic therapy (aPDT) and antibacterial blue light (aBL) have emerged as solutions for attacking dental biofilm [2,3,4,5]. APDT is an effective antibacterial tool, but investigations of its benefit in periodontitis care have generally been disappointing [6]. However, in most studies, the treatment has been applied sparsely, often only once or twice [7,8]. The treatment has appeared more effective in studies that have reported repetitive aPDT treatment [9,10,11]. Thus, the frequency of treatment can have a significant effect on the response.

Dual-light therapy is a combined application of aPDT and aBL, and it has shown significant antibacterial efficacy in biofilms and a reduction in dental plaque development [9,12,13,14]. Lumoral^®^ is a novel CE-marked medical device that provides simultaneous 405 nm aBL and 810 nm near-infrared (NIR) LED light in a mouthguard form. With indocyanine green (ICG) mouth rinse (Lumorinse^®^, Koite Health LTD, Espoo, Finland), the product offers simultaneous aBL and aPDT action. Selective adherence of ICG to dental plaque bacteria leads to targeted antibacterial activity [12,13]. In regular home use, the dual-light treatment by Lumoral^®^ reduced inflammation and inflammatory markers in peri-implant disease and has shown benefits when used to support oral hygiene at home [9,15].

Previously, the effect of aPDT treatment has been investigated only in the in-office setting. This clinical study is the first to evaluate the effectiveness of home-applied dual-light aPDT on periodontal disease symptoms and clinical appearance. We hypothesized that when added to non-surgical periodontal treatment (NSPT), regular use of potent antimicrobial treatment would reduce the microbial burden on the gingival tissue. In addition, the regularly applied near-infrared light portion in the dual light might have an additional photobiomodulation therapy (PBMT) effect on periodontal tissues. Reduction in gingival inflammation measured by bleeding on probing (BOP) was the primary outcome [16]. Thus, our statistical null hypothesis assumed no difference between the groups regarding BOP at three months.

Since this is the first large clinical trial on continuous, repetitive aPDT treatment applied at home, we performed an interim analysis to determine the clinical efficacy and potential adverse events. In this interim report, we provide the early three-month results of an ongoing randomized, prospective, controlled clinical trial designed to evaluate the effectiveness of the Lumoral^®^ treatment in periodontitis patients. (Clinical-Trials.gov Identifier: NCT05278416).

## 2. Materials and Methods

### 2.1. Study Design

This study is a single-site, randomized clinical trial designed to determine the efficacy of the regular home use of dual-light aPDT in periodontitis patients. The study protocol was approved by the ethics committee of the Hospital District of Helsinki and Uusimaa (HUS/3089/2021) and was conducted in accordance with the ethical principles of the Declaration of Helsinki and the good clinical practice (GCP) ISO 14155 international standard. All participants provided written informed consent before enrolment. For the study, patients are randomized to receive either non-surgical periodontal treatment (NSPT), including standardized hygiene instructions and electric toothbrush, and scaling and root planing (NSPT-group), or NSPT with Lumoral^®^ (NSPT + Lumoral^®^-group) treatment.

### 2.2. Sample Size

**Figure 1:**
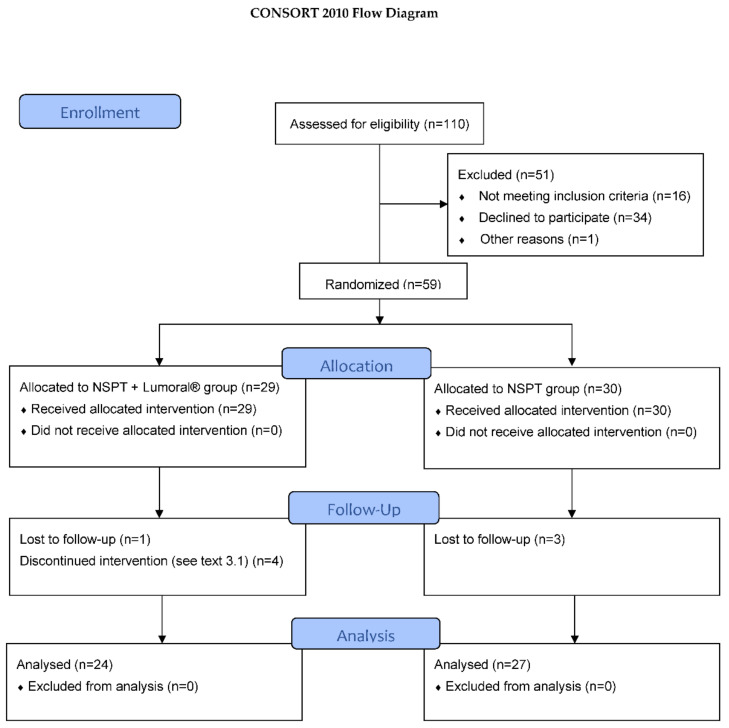
Flow diagram according to the CONSORT protocol.

This interim report includes 59 consecutive patients of a total of 200 patients that shall be recruited for the entire clinical trial. Figure 1 shows the CONSORT flow diagram reporting the enrolled, treated, and evaluated patients. For the interim analysis, there were 24 patients in the NSPT + Lumoral^®^-group and 27 in the NSPT group. Any subjects over 18 years old were eligible for recruitment according to the following inclusion and exclusion criteria.

Due to the scarcity of series in repeated antibacterial PDT use in periodontitis, the decision was made to include 200 patients in the study. The number of patients is based on previous clinical trial data of periodontal aPDT treatments showing sufficient cohorts to allow statistical calculations [17,18,19]. Power analysis for the interim analysis was calculated (SAS 9.4, SAS Institute, Cary, NC, USA) from the previous study design, assigning 5% for alpha errors and 20% for TYPE II errors (80% power). Using the allocation ratio of 1 and blind to the data, we assumed a mean of BOP at 0.48 ± 36 in the control group and 0.19 ± 0.22 in the treatment group. The resulting sample size of 20 subjects in each group encouraged us to analyze this cohort.

### 2.3. Eligibility Criteria for Study Participants

Subjects have been referred to dental hygienist treatment by dentists or through the oral health care appointment system of the City of Helsinki. Patients were recruited during their regular oral health care visits at the Metropolia University of Applied Sciences (Helsinki, Finland). Any subjects over 18 years old were eligible for recruitment according to the following inclusion and exclusion criteria.

#### 2.3.1. Inclusion Criteria

- Periodontal disease stage I–III, according to criteria the American Academy of Periodontology (AAP) with at least 2 mm interdental clinical attachment level (CAL) in the site of greatest loss;
- Age of 18–85 years;
- Presence of ≥20 teeth;
- Agreement to participate in the study and to sign a written consent form.

#### 2.3.2. Exclusion Criteria

- Untreated/uncontrollable diabetes mellitus (DM) with HbA1c ≥ 7% and HbA1c ≥ 8 if insulin-treated DM.
- Any systemic disease (e.g., wound healing dysfunctions) that could alter the progression of periodontal disease.
- Use of medicine that would affect the periodontal tissue within the last six months (antibiotics, anti-inflammatories, anticonvulsants, immunosuppressants, or calcium channel blockers, including doxycycline, bisphosphonates, and chlorhexidine).
- Periodontal treatment during the previous three months.
- Allergic to photosensitizer.
- Presence of significant physical limitations or restrictions that prohibit the hygiene procedures used in the study protocol.
- Removable major prosthesis or major orthodontic appliance.
- Current smoking or habitual use of smokeless tobacco products.
- Pregnancy or lactation.
- A need for hopeless tooth extraction or open cavities in need of immediate endodontic treatment.

### 2.4. Randomization

The randomization was performed with a sealed envelope system. Patients were randomized in a 1:1 group assignment ratio to the NSPT + Lumoral^®^ and NSPT groups. Envelopes were sealed, mixed thoroughly, and then numbered. The examiner received the sequentially numbered sealed randomization envelopes.

### 2.5. Intra-Examiner Reproducibility

All clinical measurements were performed by a single experienced examiner (S.P.). The examiner was calibrated at the study’s beginning regarding the probing pocket depth (PPD) and clinical attachment level (CAL). Using a manual probe, the examiner examined four teeth at six sites per tooth, with 24 measuring sites of the phantom head (Frasaco GmbH, Tettnang, Germany). The recordings for PPD and CAL were performed and documented in two sessions, maximum of 48 h apart. Calibration was considered acceptable if measurements at baseline and 48 h were equal to the millimeter at >85% level.

### 2.6. Clinical Procedure

Demographic data were recorded. At baseline and the 3-month follow-up visit, a complete clinical intraoral examination was performed, and clinical measurements, including BOP, visible plaque index (VPI), and PPD, were obtained.

After baseline measurements, all patients received standard anti-infective treatment. Those randomized in the NSPT + Lumoral^®^ group were given a Lumoral^®^ treatment device and Lumorinse^®^ mouth rinse to perform dual-light aPDT at home.

### 2.7. Anti-Infective Treatment

The standard anti-infective treatment included thorough cleaning (scaling and root planing, SRP) of all pockets (≥4 mm) using an ultrasound instrument (NSK Varios 750 with scalper tips G6 and G9, NSK Dental, Kanuma, Japan) with water cooling or hand instruments (LM Gracey curettes, LM-Dental Instruments, Parainen, Finland) together with cleaning powder (Perio-mate with Perio-mate powder, NSK Dental, Kanuma, Japan). In the case of asthma, RDA250 Topdent pasta cleaning was used instead.

In addition to the anti-infective treatment, standard oral hygiene instructions, including the use of an electric toothbrush, interdental brush, and dental floss, were provided to all participants. All patients received a similar electric toothbrush (Jordan AS, Oslo, Norway).

### 2.8. Clinical Measurements

All clinical measurements were recorded with the help of a graded periodontal manual probe (North Carolina 54B, Hu-Friedy Mfg. Co., LLC, Chicago, IL, USA) with a maximum force of 0.25 N for six sites (mesiobuccal, buccal, distobuccal, mesiolingual, lingual, and distolingual) of each tooth.

BOP was assessed at six sites per tooth, based on the presence or absence of gingival bleeding within 15 s after gentle probing, and it was reported as the percentage (%) of sites with positive findings of full mouth. VPI was assessed of full-mouth, with a six-point dichotomous scoring as plaque “1 present” or “0 absent”. VPI is presented as the percentage (%) of sites with positive findings. PPD was measured in millimeters from the gingival margin to the base of the periodontal pocket.

### 2.9. Dual-Light aPDT Treatment

**Figure 2:**
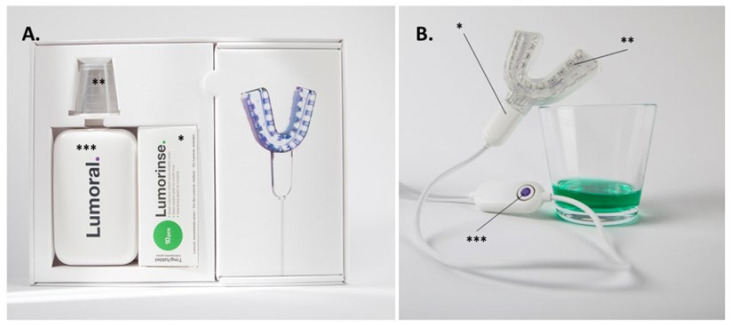
The dual-light aPDT Lumoral^®^ device. (**A**) The device includes the Lumoral^®^ light applicator and the effervescent Lumorinse^®^ tablets (*) to be dissolved in 30 mL of water with a measuring cup (**). A power source (***) for the light applicator. (**B**). The mouthpiece (*) is composed of 48 LED components (**) assembled to provide simultaneous light on both the maxillary and mandibular dental arches. Each LED component emits 405 nm and 810 nm light simultaneously. A push of the control button provides a treatment time of 10 min (***). When dissolved, the ICG in the Lumorinse^®^ tablet appears green.

The Lumoral^®^ treatment device is a CE-marked antibacterial home-use device for treating and preventing oral diseases caused by bacteria. During use, the Lumoral^®^ device provides light to activate a CE-marked mouth rinse called Lumorinse^®^ (see Figure 2). 

Patients received detailed instructions for the use of the Lumoral^®^ treatment device and Lumorinse^®^ mouth rinse tablets and were instructed to use the Lumoral^®^ treatment device and follow the protocol once a day. 

The Lumorinse^®^ mouth is an effervescent tablet with a final ICG concentration of 250 μg/mL. The mouth rinse is swished for 60 s enabling ICG to adhere to dental plaque. The light activator mouthpiece, or Lumoral^®^ treatment device, is used to simultaneously activate the ICG attached to the plaque at the lower and upper dental arches. Lumoral^®^ has 48 LED components delivering light on both dental arches. The device provides dual-light action, where 810 nm light activates IGC adhered to the bacteria, and 405 nm aBL is absorbed by the intrinsic chromophores, mainly porphyrins and flavins within the bacterial cell. After 10 min and 30 J/cm^2^ radiant exposure, the device automatically turns off. The treatment is used in adjunct to regular dental hygiene procedures, such as toothbrushing, interdental brushing, and dental floss use. 

### 2.10. Compliance and Adverse Events Reporting

Patients in the study group were asked to keep a diary of the Lumoral^®^ use and return the remaining Lumorinse^®^ tablets at the 3-month follow-up visit to determine the total usage. Compliance was calculated as a percentage of the number of tablets used per the number of treatment days. Lumoral^®^ users were also asked to observe and self-report any adverse effects of the Lumoral^®^ treatment.

### 2.11. Statistical Analysis

GraphPad software version 9.1.0 (GraphPad Software, La Jolla, CA, USA) was used to analyze the data and create the graphs. A Wilcoxon nonparametric analysis of paired groups was performed to compare the difference of continuous variables, and the Mann–Whitney test was used to compare unpaired samples. In addition, Fisher’s test was used for contingency analysis for the dichotomous variables. A *p*-value less than 0.05 was considered statistically significant. 

## 3. Results

### 3.1. Demographic Characteristics of the Patient Population

Out of 110 patients who were assessed for eligibility, 59 patients were randomized. The most common reason for unwillingness to participate was the lack of commitment due to time restrictions. The most common reasons for exclusion were current smoking, uncontrolled diabetes, less than 20 remaining teeth, or the inability to understand the study protocol due to language problems. Three patients in the NSPT group and one in the NSPT + Lumoral^®^ group did not arrive at the cut-off point at the three-month visit. Four patients in the NSPT + Lumoral^®^ group and none in the control group interrupted the study, *p* = ns., Fisher’s Test. One patient experienced excess saliva secretion and vomiting reflex during device use. Two patients felt discomfort from the warmth produced by the device. One patient did not arrive at the three-month visit and was informed about the decision to discontinue the study. There were nineteen females and six males in the NSPT + Lumoral^®^ and eighteen females and nine males in the NSPT group (*p* = ns.). The persons interrupting the study were all females. In the final analysis, 51 patients were included, see Figure 1.

### 3.2. Bleeding on Probing (BOP)

**Figure 3:**
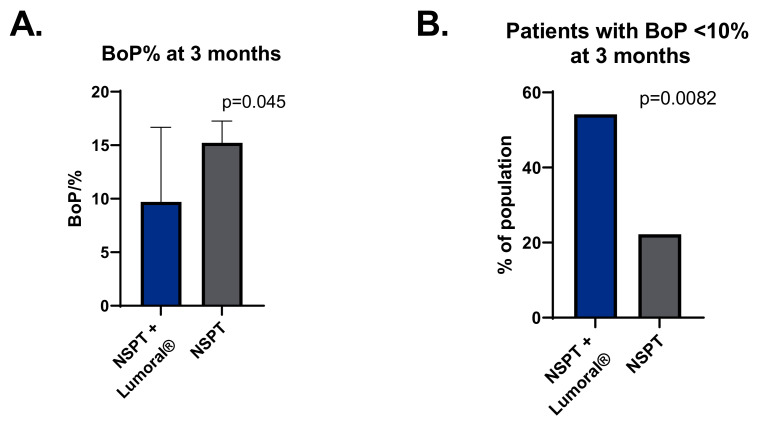
(**A**) BOP (%) at the three-month visit. (**B**) The percentage of participants with BOP < 10% at the three-month visit. BOP = bleeding on probing, NSPT = non-surgical periodontal treatment.

At the beginning of the study, the median BOP was 23.7% (range 8.0–48.5%) in the NSPT + Lumoral^®^ group and 26.5% (range 13.2–53.8%) in the NSPT group, with no statistical difference between the groups. Both groups showed a reduction in BOP at the three-month visit, but the BOP in the NSPT + Lumoral^®^ group was significantly lower (*p* = 0.045), with medians of 9.7% (range 2.8–34.5%) and 14.2% (range 5.6–35.9%), respectively. See Figure 3A. In the NSPT + Lumoral^®^ group, 54% of the patients and 22% in the NSPT group reached BOP < 10% target (*p* = 0.02), see Figure 3B.

### 3.3. Plaque Index

**Figure 4:**
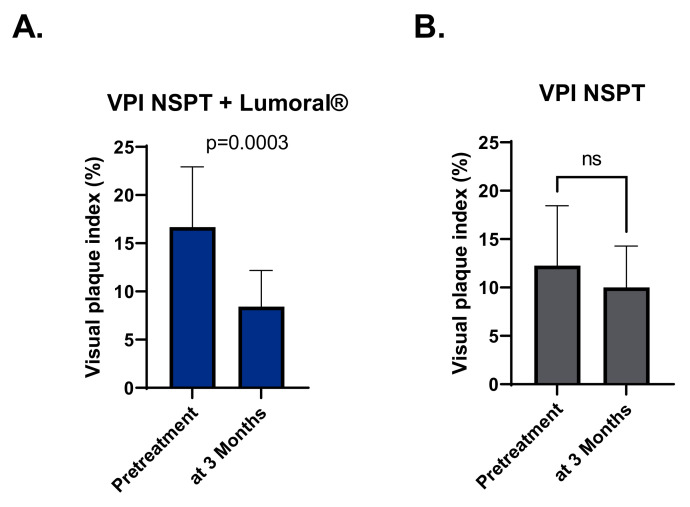
(**A**) VPI (%) in the NSPT + Lumoral^®^ group at the beginning of the study and three months. (**B**) VPI (%) in the NSPT group at the beginning of the study and three months. VPI = visual plaque index, NSPT = non-surgical periodontal treatment.

At the beginning of the study, the median VPI in the NSPT + Lumoral^®^ group was 16.67% (range 3.09–42.22%) and 12,25% (range 2.47–38.1) in the NSPT group, with no statistical difference between the groups. However, in the NSPT + Lumoral^®^ group, the VPI was significantly lower at the three-month time point with a median of 10.03% (range 1.33–31.55%), while in the NSPT group, the VPI remained statistically the same with a median of 12.26% (range 3.0–34.6%). See Figure 4.

### 3.4. Periodontal Pockets

**Figure 5:**
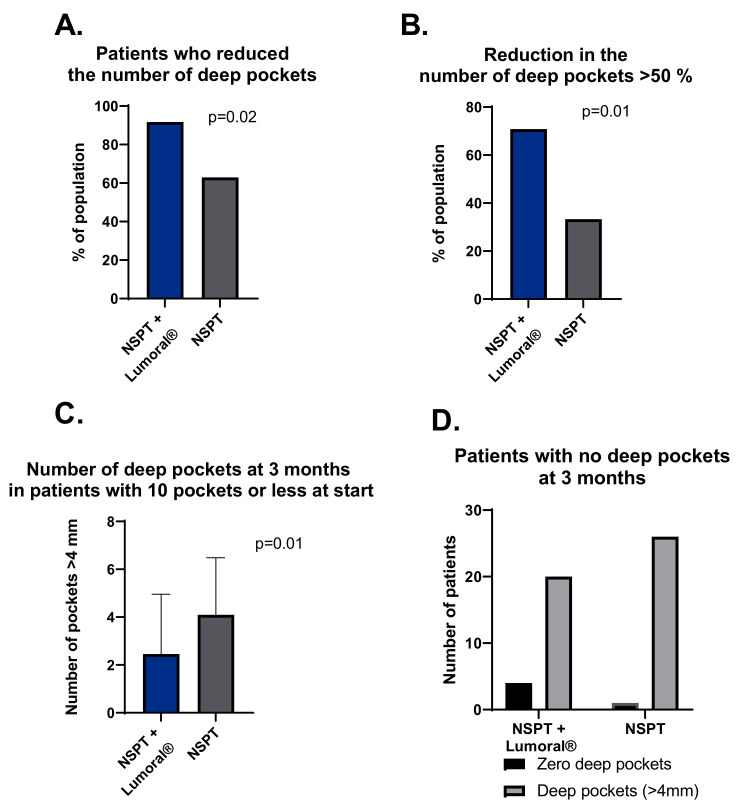
(**A**) Proportion of the patients who reduced the number of their deep periodontal pockets. (**B**) The number of patients with a reduction of 50% or more in the number of deep pockets at three months. (**C**) Number of deep pockets at three months in patients who had ten pockets or fewer at the beginning of the study. (**D**) The number of patients who presented no deep pockets at three months.

At the beginning of the study, the median number of deep periodontal pockets in the NSPT + Lumoral^®^ group was 6 (range 0–29), and in the NSPT group, a median of 7 (range 1–41) with no statistical difference between the groups. Both groups showed a significantly lower number of deep pockets at the three-month visit when the median number of deep pockets in the NSPT + Lumoral^®^ group was 3.5 (range 0–14) and in the NSPT group 5 (range 0–14). The portion of patients who reduced the number of their deep periodontal pockets was 92% in the NSPT + Lumoral^®^ group and 63% in the NSPT group (*p* = 0.02). The fraction of patients reducing the number of their deep pockets by 50% or more was 71% in the NSPT + Lumoral^®^ group and 33% in the NSPT group (*p* = 0.01). Patients with less than ten deep pockets at the beginning had a median of 1 (range 0–8) pocket at three months in the NSPT + Lumoral^®^ group and a median of 4 (0–9) in the NSPT group (*p* = 0.01), while no statistical difference initially. At three months, four patients with no deep pockets were found in the NSPT + Lumoral^®^ group and one in the NSPT group. See Figure 5. 

### 3.5. Compliance and Adverse Events

The compliance to the Lumoral^®^ use in those who completed the three-month surveillance was a median of 88%, ranging from 24% to 100%. There were no device-related serious adverse events. Device-related adverse events included a sensation of warming or numbness in the mouth or tongue (4) and excess salivary production (2). In addition, two patients discontinued the device use due to the sensations (7%), and two patients due to other reasons (7%).

## 4. Discussion

This randomized and controlled clinical study was designed to evaluate the clinical benefit of adjunctive antibacterial dual-light in periodontitis treatment. Here we provide an interim analysis of 51 patients completing the three-month follow-up. Both groups received an intervention to improve oral hygiene and toothbrushes for standardized oral hygiene. Dual-light aPDT was applied at home with a Lumoral^®^ device. As the primary outcome, adjunctive Lumoral^®^ use resulted in lower BOP than NSPT alone at three months compared to the baseline. Lumoral^®^ use also resulted in a significantly larger number of patients reaching the target value of <10% BOP, or periodontal health, compared to the NSPT alone. The statistical null hypothesis of no difference between the groups regarding BOP at three months is rejected based on current results. In addition, adjunctive Lumoral^®^ treatment improved oral hygiene, while in the NSPT group, we observed no change. As expected, both treatments led to statistically significant improvements at three months in BOP and PPD. However, additional Lumoral^®^ treatment significantly reduced the number of deep periodontal pockets compared to the NSPT alone. In that regard, all but two, or over 9/10, of the patients in the Lumoral^®^ group reduced the number of deep periodontal pockets. Moreover, over two thirds of the patients in the Lumoral^®^ group reduced the number of their deep pockets by 50% or more during the three months. In the NSPT group, less than two thirds of patients reduced the number of deep pockets, and one third of patients reduced the number of deep periodontal pockets by 50% or more. These results are in line with our previously reported effects of regular dual-light treatment, where a significant reduction in aMMP-8, BOP, and VPI were seen in periodontal patients with intensive, regular, up to twice daily, Lumoral^®^ use [9]. Similarly, a recently published case report showed a significant improvement in periodontal disease status after regular Lumoral^®^ use in a patient with difficulties in the mechanical performance of oral hygiene at home [15]. Furthermore, the previously published reports of in-office applied ICG-mediated aPDT have shown effectiveness as adjunctive treatment in periodontitis care [18,19,20,21]. 

In 2020, the European Federation of Periodontology (EFP) published its S3 Level Clinical Practice Guideline (CPG) for the treatment of Stage I–III periodontitis [22]. The guideline is based on a pre-established stepwise approach to therapy. The treatment starts with behavioral changes for improved control of supragingival biofilm and gingival inflammation, including risk factor control, before heading to the supra- and sub-gingival instrumentation. Additionally, case to case, different types of periodontal surgical interventions and supportive periodontal care can add benefits. The protocol in the present study was designed to accommodate the guideline structure. The guidance in oral hygiene and the use of electric toothbrushes in both groups aimed to improve home treatment as much as possible. The additional cleaning of the sub- and supragingival plaque in the clinic further reduced the effect of the microbial burden on the periodontal tissues. Most patients were in maintenance therapy, with a median number of deep pockets at 6–7. Furthermore, the recruitment process can select more eligible people to care for themselves. Despite reasonable disease control, the significant improvement in periodontal status with adjunctive Lumoral^®^ treatment is exciting.

The antimicrobial effect of aPDT is mainly based on the principle that visible light activates an externally applied photosensitizer, producing reactive oxygen species (ROS) that kill bacteria indiscriminately through oxidative bursts [2]. Antimicrobial blue light (aBL) is based on the same principle, but the photosensitizers of the latter process are internal molecules in bacteria, such as porphyrins and flavins [3,4]. We have investigated the effect of combined aPDT and aBL against different bacterial biofilms using an ICG photosensitizer. The simultaneous application of aBL has significantly increased the bactericidal impact compared to the aPDT alone [12,13,14,23]. However, the ability of the adjunctive Lumoral^®^ treatment to provide an antibacterial effect in the periodontal pocket is not known. Although the 810 nm light has a good tissue penetration to provide transgingival light energy, the concentration of the ICG in the periodontal pocket after mouth rinsing remains questionable.

Indocyanine green (ICG) is a widely used aPDT photosensitizer in dentistry due to its low toxicity, non-ionizing properties, water solubility, and light absorption at near-infrared (NIR) wavelengths, which have good tissue penetration [12,24]. Several studies have shown the efficacy of NIR 810 nm/ICG aPDT as an adjunctive periodontal treatment. In a recent review, Moro et al. found ICG–mediated aPDT superior to other aPDT methods [25]. In another systematic review, Bashir et al. evaluated the efficacy of adjunctive ICG-based aPDT in periodontal patients. They found a mean additional pocket depth reduction of 1.17 mm at three months and a mean additional reduction of 1.06 mm at six months compared to the SRP alone [26]. However, a general issue in adjunctive aPDT studies is the heterogeneity of the administration protocols and, maybe even more importantly, the low frequency of the treatment [27]. Multiple obstacles are likely to arise when the treatment requires repeated visits to a dentist’s office, where special equipment and expertise are needed. Auspiciously, rapid development in light-emitting diode (LED) technology has allowed the development of personal products for light applications used at home. In a home setting, the aPDT treatment can be self-administered by the patients on a more regular and frequent basis. The benefits of continued use of aPDT are not evident in the current literature, and this study provides new information.

Non-surgical periodontal therapy as the standard treatment reduces the bacterial burden and microbe-induced inflammatory response, creating a favorable environment for reattachment. However, the debridement simultaneously causes tissue trauma in the inflamed periodontal tissues, where healing occurs. The dual-light aPDT promotes not only the antibacterial action to sustain the oral hygiene reached but, potentially, an effect of PBMT, possibly leading to improved wound healing. PBMT is a complex process where the photon energy is absorbed within tissues, mainly in cytochrome-c-oxidase, and leads to induced ATP production, DNA and RNA synthesis, nitric oxide production, and modification of cellular membrane activity [28]. PBMT has been most prominently used in oral mucositis and has been recognized in the treatment recommendations [29]. In a meta-analysis by Peng et al., PBMT has been declared the most effective treatment for oral mucositis, with a probability of 95.8% [30]. However, the efficacy of PBMT in periodontitis remains debatable due to the limited available literature. However, again, the treatment frequency has been low in the studies regarding adjunctive periodontal PBMT. In a systematic review of Dalvi et al., only two study protocols described 5–10 treatment sessions [28]. Although Lumoral^®^ treatment can provide PBMT regularly, this study’s effect of photobiomodulation in the healing process remains debatable.

The present study has clinical implications for the future. Compliance with regular mechanical cleaning at home is the most crucial factor in periodontitis treatment. However, because even the best toothbrushes remove only about 65% of dental plaque, even those who pragmatically clean their teeth have significant room for improvement regarding oral hygiene. Antibacterial PDT exerts extremely potential efficacy, and now for the first time, the aPDT is available in the home environment. Furthermore, the dual-light evolution makes aPDT even more effective. Interestingly, the dual-light aPDT provides a targeted antibacterial effect on the plaque, which can benefit in preserving the oral bacterial flora. Reduction in side effects is welcome when adjunctive antiseptic products are used in managing gingival diseases [31].

There are certain limitations in this study. These interim results with a three-month surveillance period are not long enough to give certainty as to the longer-term benefit of the device’s use. On the other hand, using the device can motivate the user for improved hygiene. A sham device would be optional for the control group to provide an even more reliable protocol. However, providing a light-providing sham device is not a simple task. Photobiomodulation is supposed to act through the cytochrome-c-oxidase enzyme. Because of the broad absorption spectrum of the cytochrome-c-oxidase, avoiding the photobiomodulation effect could need a separate investigation. Finally, even though the number of subjects in this report resembles the population size used in similar studies [17,18,19], a larger population would provide more reliability in the statistical analysis.

## 5. Conclusions

These three-month results suggest that regular adjunctive dual-light aPDT therapy applied with a Lumoral^®^ device can effectively improve NSPT results. However, the six-month results are warranted to define the effectiveness of the treatment further.

## Data Availability

The data supporting the results reported here are available from the corresponding author upon request.
